# Group metacognitive therapy for students with anxiety: an uncontrolled pre-post study of feasibility and associated outcomes

**DOI:** 10.3389/fpsyt.2026.1766026

**Published:** 2026-03-26

**Authors:** Hedda Friis Jørgensen, Emma Karin Helvig, Hilde Schei, Shirley Stormyren, Elin Steffensen, Sverre Urnes Johnson

**Affiliations:** 1 Department of Psychology, University of Oslo, Oslo, Norway; 2 Student Welfare Organization in Oslo (SiO), Oslo, Norway; 3 Modum Bad Psychiatric Center, Vikersund, Norway; 4 Department of Psychology, Harvard University, Cambridge, MA, United States

**Keywords:** anxiety disorders, psychotherapy, group, metacognition, depression, clinical relevance, MCT

## Abstract

**Background:**

Group-based Metacognitive Therapy (gMCT) has shown promising results for anxiety, but research specifically targeting student populations is limited. By addressing maladaptive metacognitions, gMCT aims to reduce core symptoms such as anxiety, excessive worry, and rumination. More research is needed in university settings to determine whether gMCT can be successfully applied to students. Method: We investigated the feasibility and examined a gMCT treatment course with an uncontrolled pre-post design with a 3-month booster follow-up. The design reflects the available data material and the limitations of the real-world clinic setting. Participants were students with a score >=10 on GAD-7 assessment, or diagnosed with anxiety disorders, including Generalized Anxiety Disorder (GAD), Social Anxiety Disorder (SAD), and Panic Disorder (PD). Patients presenting with additional conditions that would necessitate alternative interventions/treatment were excluded and not eligible for this study. The generic MCT treatment model was delivered as part of routine care by therapists at the Oslo Student Welfare Organization (SiO Helse) who had relevant MCT training. Data material was collected at pre-treatment, post-treatment, and booster follow-ups. Feasibility was assessed using attendance, dropout, and number of completers. Missing data were handled using multilevel modelling and mean imputation at the item level. The primary outcome was GAD-7, and depressive symptoms (secondary outcome) with the PHQ-9. Worry and Metacognitive beliefs were assessed. Analyses included paired sample t-tests, clinically significant change analysis, and multilevel modelling (MLM) to evaluate change over time.

**Results:**

Treatment adherence was high, with participants attending an average of 7.1 out of 8 sessions. Significant reductions were observed across all measures from pre- to post-assessment, with large effect sizes for the primary outcome (GAD-7) and for worry (CAS-1), negative metacognitions (MCQ-30), and depressive symptoms (PHQ-9). These improvements were maintained at the 3-month booster follow-up.

**Conclusion:**

gMCT is feasible for treating student populations with anxiety and depression symptoms. Overall, 77.3% of participants improved or recovered after post-assessment. The results should be cautiously interpreted due to the single-site setting, few patients and short follow-up period, but suggest that other student welfare organizations may benefit from implementing gMCT to treat anxiety- and worry-related problems in student populations.

## 1 Introduction

Mental health among university students is a significant global public health concern, with 31.4% of first-year students screening positive for at least one common mental disorder. This prevalence exceeds what’s typically observed in the general population. Anxiety and mood disorders are the most common problems, with 24.5% of students meeting criteria for an anxiety disorder and 25% for depression (1). Untreated psychological disorders have harmful consequences for the students’ academic and social function, and may affect future career opportunities and academic progression if mental health difficulties hinder degree completion (2). Despite the availability of evidence−based treatments, a substantial treatment gap exists among university students. Individual therapy alone is often not a cost−effective or sustainable response to the growing demand. Student welfare services therefore need to explore scalable approaches, such as group metacognitive therapy (3, 4). While individual MCT is supported by a substantial evidence base (5, 6), group metacognitive therapy (g-MCT) may represent a potentially cost-effective alternative (7), requiring significantly fewer therapist hours per patient to achieve comparable clinical outcomes. Beyond economic efficiency, g-MCT facilitates unique therapeutic factors such as peer normalization, which helps reduce stigma, and vicarious learning, as patients with different worry contents can help each other identify shared maladaptive metacognitive beliefs and coping strategies (8, 9).

Metacognitive therapy (MCT) builds upon the self-regulatory executive function model (S-REF model) (10), which serves as the theoretical foundation for this study. The S-REF model conceptualizes emotional distress as being maintained by a multi-level cognitive architecture consisting of automatic processing, attentionally demanding voluntary processing, and stored knowledge or self-beliefs (11). Within this framework, metacognition is described as a higher-rank cognition that controls, monitors, and interprets thinking across situations (11, 12). In essence, metacognitions are thoughts about thoughts and guide our cognition through three interrelated components: Metacognitive knowledge and beliefs (positive or negative), experiences, and strategies (3, 11).

When metacognitive processes become maladaptive, they result in a dysfunctional thinking pattern known as Cognitive Attentional Syndrome (CAS). The CAS is characterized by uncontrollable and excessive worry and rumination, heightened self-focus attention, and fixated attention toward threat stimuli (3). This syndrome is considered problematic because it creates a repetitive and negative self-regulatory cycle that depletes cognitive resources necessary for disconfirming dysfunctional beliefs. Through rumination, the CAS continuously primes dysfunctional self-beliefs, increasing the likelihood of distress-conforming information and obstructing mechanisms necessary for adaptive emotional processing (10). A substantial body of research has demonstrated the efficacy of MCT in treating anxiety disorders, as well as depressive disorders (3, 5, 11, 13). Additionally, MCT interventions have been found to influence metacognitive appraisals and result in high recovery rates at follow-up (6, 14).

The tendency for some students to develop the CAS may be explained by prevalent metacognitive profiles among university students and the role of negative metacognitive beliefs acting as transdiagnostic vulnerability factors. Recent research suggests that negative metacognitive beliefs, specifically regarding the uncontrollability and danger of worry, serve as a critical mediator between adverse childhood experiences and current mental well-being in young adults. These early negative life experiences can trigger maladaptive metacognitive processes that activate the CAS, making students more susceptible to prolonged emotional distress (15). Furthermore, student-specific stressors, such as academic pressure or future-related worry, may elicit automatic negative thoughts. Students may experience such thoughts as difficult to handle if their metacognitive regulatory capacity is limited and find themself struggling to handle the anxiety and stress associated with these thoughts. This increases psychological vulnerability and is associated with modern challenges such as fear of being without reliable connectivity (nomophobia) (16). Difficulties in metacognitive regulation can also impair a student’s ability to cope with everyday challenges, which can temporarily reduce motivation and academic persistence. However, metacognitive interventions aimed to increase awareness and control over cognitive processes may effectively strengthen academic persistence, helping students maintain their performance and well-being despite stressful academic conditions (17).

MCT is a transdiagnostic approach, focusing on universal factors across pathologies, such as excessive worry and rumination, rather than focusing on specific symptoms. (18). This is particularly beneficial given the high rates of comorbidity observed in clinical settings. For instance, approximately 67% of those with depression also have a comorbid anxiety, while 63% of those with anxiety experience concurrent depression (19–21). Empirical findings show that dysfunctional metacognitive beliefs are strongly associated with symptoms of both depression and anxiety (22).

While robust evidence supports the efficacy of gMCT across various adult clinical populations, reporting large effect sizes for symptoms of anxiety, depression, and dysfunctional metacognition (8, 14, 23–25); there remains a notable deficit of empirical research focused specifically on university students Hence, there is still considerable uncertainty regarding optimal treatment strategies for the student demographic (26). Consequently, further research is required to evaluate the applicability and associated outcomes of gMCT within student-specific clinical environments.

A feasibility assessment examines whether an intervention can be delivered as intended in a study. That is a process-focused assessment that aims to reduce uncertainty by evaluating practical aspects of delivery (27).

This study aims to evaluate the feasibility and associated symptom changes in an ongoing gMCT treatment conducted by clinical therapists at the Oslo Student Welfare Organization (SiO Helse) as part of routine care. We investigated this by the following four research questions:

1. What is the feasibility of this intervention?

2. What are the changes associated with gMCT in primarily anxiety, secondly depression, and metacognitions among students with anxiety and worry difficulties?

3. What are the changes associated with gMCT in worry among students?

4. What long-term changes are associated with the treatment?

## 2 Methods

### 2.1 Participants

Participants were recruited from an outpatient clinic operated by the Student Welfare Organization in Oslo (SiO Helse). A total of 61 patients, aged 19 to 31 (90.2% female), were eligible for this study using an uncontrolled pre-post design with a 3-month booster follow-up (see **Table 1** for descriptions). The design was determined by the nature of the data collected and made available by SiO Helse. Due to SiO Helse being an out-patient clinic operating under ordinary clinical practice, no control-group data were available.

**Table 1:** Sample descriptives.

Gender and age group		Number	Percentage of sample
			(%)
Male		6	9.8
Female		55	90.2
19-25		50	82
26-31		11	18

Students who receive treatment at SiO helse are self-referring. Students who report issues related to symptoms of anxiety in a broad range were recruited to an assessment with one of the group therapists. The intention was to recruit broadly among students with anxiety and worrying symptoms, since it’s one of the most common reasons for seeking help. The main idea was to include students who experienced anxiety and worrying as their major psychological need for treatment. The clinical interview included a brief review of the patients’ psychiatric history, trauma, family/relationships, reported symptoms of GAD, SAD, and PD, and day-to-day function. Parts of Mini (D, F, N) were used to screen for current diagnosis, and GAD 7 and PHQ 9 were also part of the assessment. Inclusion criteria were designed to be liberal and based upon the criteria used by SiO Helse for treatment screening. Inclusion criteria were, but not exclusive to, (a) patients acknowledging worry as their main problem, or existing diagnosis of panic disorder, social anxiety disorder, or generalized anxiety disorder (GAD), (b) participants scored >=10 on GAD-7 assessment, (c) currently university students in Oslo, over 18 years old, Norwegian speaking, and provide informed consent, (d) assessed with a high probability of benefiting from treatment and presented high motivation for treatment. Exclusion criteria were severe depression, PTSD, severe OCD, and issues related to personality disorders. Exclusion criteria were only assessed through clinical evaluation.

**Figure 1:**
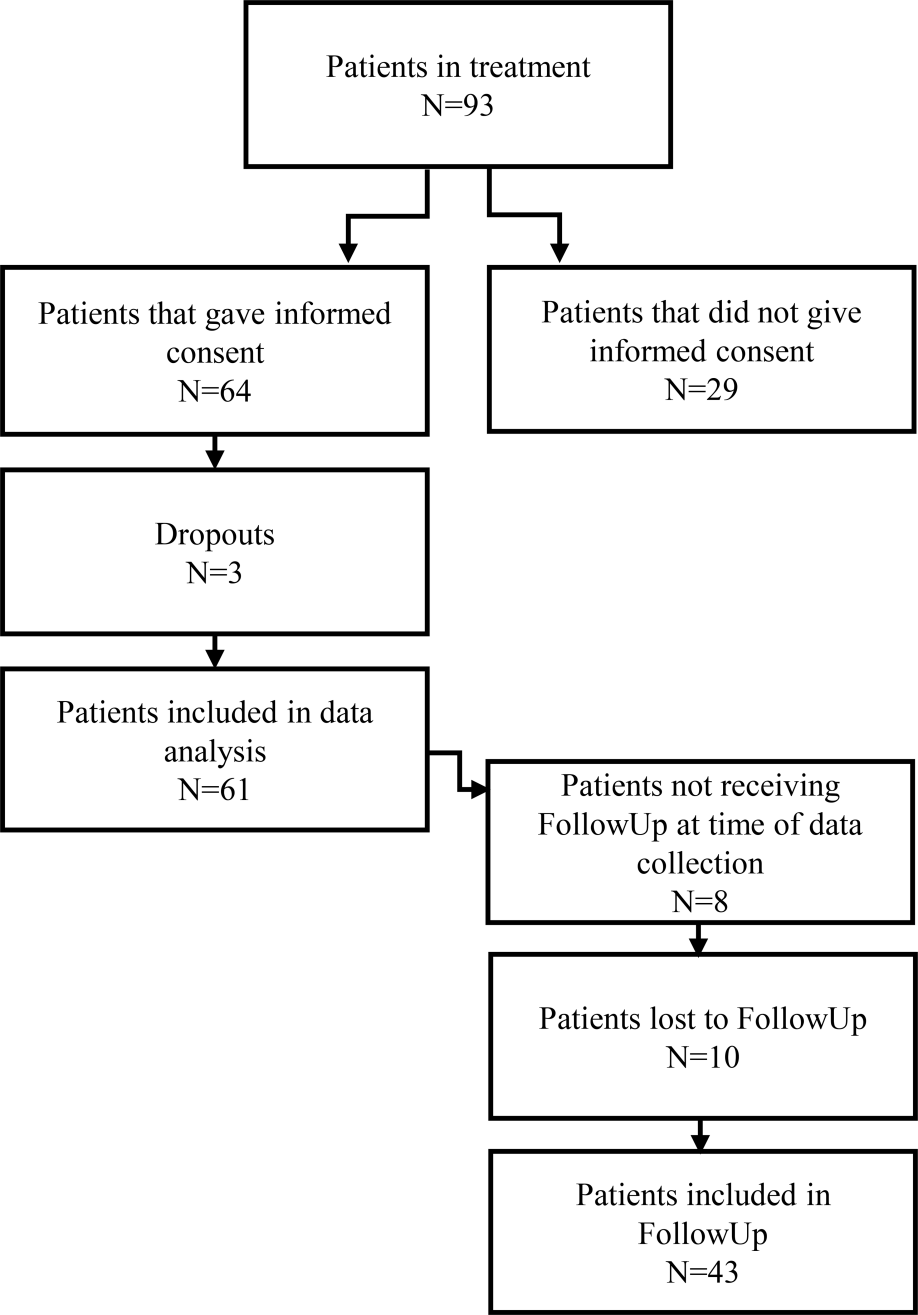
*Patient flow-chart*. A flowchart illustrating who was included or excluded from data analysis.

In general, 42% of students who attend the assessment interview start in the treatment group. The percentage varies by semester, since more than half of students either fail to meet eligibility criteria, cannot follow the treatment schedule, or prefer individual treatment; hence about 220 students were evaluated for the current study. As illustrated in **Figure 1**, 93 patients received treatment at SiO Helse and 64 patients provided informed consent. Three were flagged as dropouts, leaving a data sample of N = 61. Dropouts were flagged if they failed to complete five sessions or more. The dropouts’ baseline was similar to the completers for GAD-7 (Dropout=12;12;13, completers mean=13). For PHQ, the dropouts baseline deviated both ways from the completers’ mean (Dropouts=15;15;7, completers mean= 11). At the time of data analysis, one treatment group had not completed follow-up measures, leaving the sample at follow-up N = 53.

Six patients were included by the therapist’s clinical evaluation despite a score <10 on GAD-7. These exceptions reflect the real-world setting and psychologists’ discretion in addition to the patients’ overall condition, level of functioning and likelihood of benefiting from treatment. An additional sensitivity analysis was employed to investigate the possible impact this could have on our primary outcome, anxiety. In the current study, we assess feasibility through the use of treatment attendance and dropout rates. Attendance was operationalised as the number of sessions attended, with treatment completion defined as attending at least five out of eight sessions. Dropout was defined as discontinuation before session five.

### 2.2 Outcome measures

#### 2.2.1 anxiety (primary outcome)

Anxiety symptoms were measured using the GAD-7 questionnaire. This is designed to assess the presence and severity of Generalized Anxiety Disorder (GAD). It comprises seven items, each scored on a scale from zero to three. GAD-7 divides test scores into three categories. The scale has three cut-off points: 5 (mild), 10 (moderate), and 15 (severe) (28).

#### 2.2.2 Metacognitions (secondary outcome)

MCQ-30 consists of 30 items measuring multiple metacognitive parameters. It is a shorter version of the MCQ-65 assessment. The 30 questions have a scale from one to four (3). For MCQ-30, changes in scores are used as indicators of the treatment’s ability to change the metacognitive mechanisms. Specifically, they measure the participants’ thoughts and attitudes toward their thoughts and the frequency of rumination. The obtained scores serve as indicators of the levels of maladaptive metacognitions; higher scores correspond to higher levels of maladaptive metacognitions, while lower scores imply lower levels (29).

#### 2.2.3 Depression (secondary outcome)

The Patient Health Questionnaire-9 (PHQ-9) measures the severity of depression symptoms. The total scores can be categorized into four categories: scores 5-9 indicate mild symptoms of depression, 10-14 indicate moderate symptoms of depression, 15-19 indicate fairly severe symptoms of depression, and 20-27 indicate severe symptoms of depression (30).

#### 2.2.4 Worry (secondary outcome)

CAS-1 monitors cognitive attentional syndrome and positive and negative underlying metacognitive beliefs. It consists of 16 items divided into three categories. The first three questions (8 items) measure metacognitive strategies. The next four items measure negative metacognitive beliefs, while the last four items measure positive beliefs (22).

### 2.3 Treatment

The treatment consisted of group metacognitive therapy led by two clinical psychologist specialists trained in MCT. No formal fidelity instrument was used, which can be a limitation in this study. The group leaders did, however, receive supervision from an experienced MCT-therapist and professor (SUJ) and followed a standardized treatment manual. Group sizes were small, with 5 to 8 participants per group. The sessions were weekly for 8 weeks and lasted 90 minutes using the generic MCT model. The treatment protocol adheres to a predetermined schedule, outlining specific topics and objectives for each session. The sessions involved targeted exercises led by therapists, followed by reflections, organized both in pairs, in triads, or collectively, during which participants explored and discussed their experiences. Participants were also assigned homework tasks and participated in behavioral experiments as part of the therapeutic process. In addition, the intervention utilized CAS-1 to closely monitor individual progress and CAS at every session. This gives valuable clinical insight and feedback from patients after every session and makes it possible for the therapists to review how the treatment is progressing every week.

### 2.4 Data analysis

The data material was collected at four time points for GAD-7, PHQ-9 and MCQ-30: screening interview (pre-), session four (medio-), session eight (post-treatment), and follow-up 2-3 months after the last treatment session (booster). In addition, CAS-1 was collected as part of treatment at every treatment session, giving nine time points. Feasibility was assessed using attendance, completion, and dropout.

To assess changes associated with treatment, a paired-samples t-test was employed to analyse mean score changes from pre- to post-treatment and from pre-treatment to booster. Cohen’s d was used for analysing effect size and assessing the impact of treatment. Cohen’s d categorizes effects as 0.2=small, 0.5=medium, or 0.8=large. Reduction in symptoms results in a positive d value.

In the present study, MLM was employed to address missing data arising at the level of repeated assessments. That is, instances in which participants did not attend one or more measurement occasions (e.g., session 4, session 7, or booster follow-up). Overall, 9% were missing at the test level (entire assessment time points), and 0.7% of data were missing at the item level (individual questionnaire items). Because the proportion of missing data at the item level was very small, mean imputation was applied to those specific items prior to analysis. Missing data at the test level, however, were handled directly by MLM through Maximum Likelihood (ML) estimation. ML estimates the model’s parameters rather than discarding incomplete observations or imputing missing values. ML treats the missing data as unobserved variables, leaving only observed (i.e., sampled) data (31). In addition, MLM provides a more complex and nuanced analysis, which is particularly advantageous in the present study as it includes repeated measures across multiple time points and a relatively large sample with considerable individual variation (32). Fixed effects are used to investigate the group-level average. Random effects make it possible to account for systematic individual variation (32).

To investigate improvement for all measures, the Jacobsen-Truax method for clinical significance (33) was used. This approach is a clinically relevant perspective. It classifies changes into four categories: ‘recovered’, ‘improved’, ‘unchanged’, and ‘deteriorated’. For GAD-7, reliable change is ± 4, and the range of normal functioning is <8 (34). For PHQ-9, reliable change is ± 6, and the range of normal functioning is <7. When statistically estimating clinical significance for GAD-7 and PHQ-9, participants below cut-off at pre-treatment were not included since they would not show meaningful change from a clinical baseline.

## Results

The following are the results of the current study sorted by the research questions.

### 3.1 Research question one: what is the feasibility of this intervention?

Uptake showed that the intervention was delivered to 42% of the patients who completed a screening interview. In total, 220 students were assessed for eligibility during the study period.

As **Figure 1** illustrates, 93 patients received gMCT at SiO Helse, and 64 patients provided informed consent to participate in the current study. Recruitment feasibility was 68.8%, which reflects the number of patients receiving treatment who consented to participate in the study. Three patients were classified as dropouts as they did not meet the criteria for intervention completion. This reflects a 95.3% treatment retention. As a result, the analysed sample for this study is N = 61. Data collection efficiency was high at post-treatment, with data provided by 59 patients (96.7%). At follow−up, 43 patients provided data (70.5%). This reduction was partly due to one full group not having completed the follow−up assessment. See **Table 2** for the means and standard deviations at the different time points.

**Table 2:** Means and standard deviations for outcome measures.

Measure	Pre-treatment	Post-treatment	Follow Up
	(*N* = 61)	(*N* = 59)	(*N* = 43)
GAD-7 (M(SD))	13.12(2.86)	7.28(3.40)	7.33(3.88)
PHQ-9 (M(SD))	10.73(4.42)	7.88(5.11)	6.70(4.75)
MCQ-30 (M(SD))	69.49(10.94)	52.02(12.31)	50.06(18.30)

M, mean; SD, standard deviation. GAD-7, generalized anxiety disorder-7(primary outcome); PHQ-9, patient health questionnaire-9(secondary outcome); MCQ-30, metacognitions questionnaire-30.

### 3.2 Research question two: what are the changes associated with gMCT in primarily anxiety symptoms, secondly depression symptoms, and metacognitions among students with anxiety and worry difficulties?

The results from paired sample t-test, as presented in **Table 3**, observed that for anxiety symptoms there was a significant decrease from pre-treatment to post-treatment with a large effect size (*t*(58)=13.17; *p*=<.001; Cohen’s *d* = 1.71; 95% CI [1.30, 2.11]). Further, the results reflect a significant decrease in the secondary outcome, depression symptoms, from pre-treatment to post-treatment with a moderate to large effect size (*t*(58); *p*=<.001; Cohen’s *d* = 0.65; 95% CI [0.36, 0.93]). In addition, findings reflect a significant reduction in metacognitions from pre-treatment to post-treatment and that this reduction was a large effect size (*t*(58)=10.35; *p*=<.001; Cohen’s *d* = 1.34; 95% CI [0.99, 1.69]). According to this, all measurements observed a significant reduction after post-assessment. Effect sizes were also large, robust, and significant for all three outcome measures.

**Table 3:** Paired sample t-test results.

Measure and time point	t	df	Sig. (two-tailed)	Cohen’s d	95%	CI
					Lower	Upper
GAD-7						
Pre-Post	13.17	58	<.001	1.71	1.3	2.11
Pre-followUp	8.75	42	<.001	1.33	0.91	1.74
PHQ-9						
Pre-Post	5.01	58	<.001	0.65	0.36	0.93
Pre-followUp	6.3	42	<.001	0.96	0.59	1.32
MCQ-30						
Pre-Post	10.35	58	<.001	1.34	0.99	1.69
Pre-followUp	6.87	42	<.001	1.04	0.67	1.41
CASstr						
Pre-Post	15.6	58	<.001	2.03	1.58	2.48
Pre-followUp	8.4	42	<.001	1.28	0.87	1.68
CASneg						
Pre-Post	8.56	58	<.001	1.11	0.79	1.44
Pre-followUp	7.14	42	<.001	1.09	0.71	1.46
CASpos						
Pre-Post	10.27	58	<.001	1.34	0.98	1.69
Pre-followUp	9.9	42	<.001	1.51	1.07	1.95

GAD-7, generalized anxiety disorder-7(primary outcome); PHQ-9, patient health questionnaire-9(secondary outcome); MCQ-30, metacognitions questionnaire-30; CASstr, CAS-1 strategies; CASneg, CAS-1 negative assumptions; CASpos, CAS-1 positive assumptions; t, t-value; df, degrees of freedom; Sig., significance value (p-value); 95% CI, 95% confidence interval.

The MLM analysis, presented in **Table 4**, strengthens these findings from paired sample t-tests. It shows a significant negative estimate from pre-treatment to post-treatment for anxiety symptoms (Estimate=-5.83; SE = 0.41; df=78.35; *p*=<.001; 95% CI [-6.66, -5.00]). For depression symptoms, there was a significant negative mean difference from pre-treatment to post-treatment (Estimate=-2.84; SE = 0.53; df=84.44; *p*=<.001; 95% CI [-3.90, -1.79]), as a non-targeted, secondary outcome. Further, the results show a significant negative mean difference from pre-treatment to post-treatment for MCQ-30 measurement (Estimate=-17.46; SE = 1.53; df=80.77; *p*=<.001; 95% CI [-20.51, -14.41]).

**Table 4:** Pairwise comparison MLM.

Measure and time point	Estimate (SE)	df	Sig.	95% CI	
				Lower	Upper
GAD					
Pre-Post	-5.83(0.41)	78.35	<.001	-6.66	-5
Pre-followUp	-5.78(0.57)	52.44	<.001	-6.94	-4.62
PHQ					
Pre-Post	-2.84(0.53)	84.44	<.001	-3.9	-1.79
Pre-followUp	-4.02(0.64)	59.51	<.001	-5.32	-2.72
MCQ					
Pre-Post	-17.46(1.53)	80.77	<.001	-20.51	-14.41
Pre-followUp	-19.43(2.37)	73.94	<.001	-24.17	-14.69

GAD-7, generalized anxiety disorder-7(primary outcome); PHQ-9, patient health questionnaire-9(secondary outcome); MCQ-30, metacognitions questionnaire-30; SE, standard error; df, degrees of freedom; Sig., significance value (p-value); 95% CI, 95% confidence interval.

### 3.3 Research question three: what are the changes associated with gMCT in worry among students?

**Figure 2:**
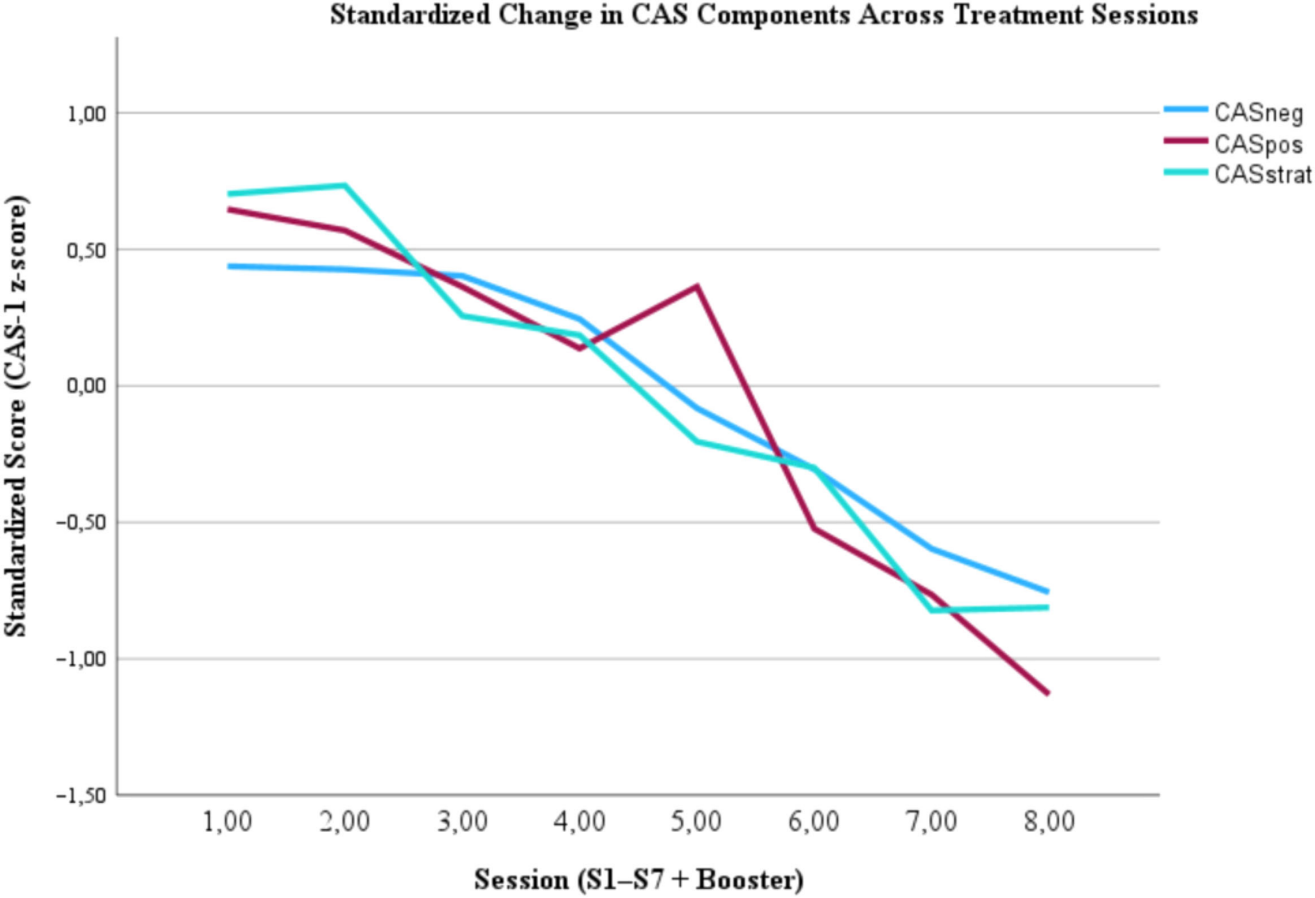
*Standardized development of CAS-1 components*. CASpos, Cognitive attentional syndrome positive; CASneg=, Cognitive attentional syndrome negative; CASstrat, Cognitive attentional syndrome strategies.

For worry, the results in **Table 3** for CAS-1 described a significant decrease in CAS strategies from pre-treatment to post-treatment (*t*(58)=15.60; *p*=<.001; Cohen’s *d* = 2.03; 95% CI [1.58, 2.48]) with a large effect size. This decrease continues from pre-treatment to follow-up (*t*(42)=8.40; *p*=<.001; Cohen’s *d* = 1.28; 95% CI [0.87, 1.68]). Further, the results also describe a significant reduction in CAS negative assumptions from pre-treatment to post-treatment (*t*(58)=8.56; *p*=<.001; Cohen’s *d* = 1.11; 95% CI [0.79, 1.44]). This reduction was persistent in the follow-up measurement (*t*(42)=7.14; *p*=<.001; Cohen’s *d* = 1.09; 95% CI [0.71, 1.46]). There was a significant reduction in CAS positive assumptions from pre-treatment to post-treatment (*t*(58)=10.27, *p*=<.001; Cohen’s *d* = 1.34, 95% CI [0.98, 1.69]). This improvement persisted in the follow-up measurement (*t*(42)=9.90; *p*=<.001; Cohen’s *d* = 1.51; 95% CI [1.07, 1.95]). These findings are visualized in **Figure 2**.

### 3.4 Research question four: what long-term changes are associated with the treatment?

For anxiety, the paired-sample t-test shows that there is a significant reduction in symptoms from pre-treatment to follow-up with a large effect size (*t*(42)=8.75; *p*=<.001; Cohen’s *d* = 1.33; 95% CI [0.91, 1.74]). For the secondary outcome, the reduction in depression symptoms was still present with a large effect size from pre-treatment to follow-up measure(*t*(42)=6.30; *p*=<.001; Cohen’s *d* = 0.96; 95% CI [0.59, 1.32]). Additionally, the results for metacognitions show that further reduction was present at follow-up with a large effect size (*t*(42)=6.87; *p*=<.001; Cohen’s *d* = 1.04; 95% CI [0.67, 1.41]). For all three measures, this reduction is robust and lasting until follow-up.

The MLM continues to support the findings from the paired sample t-test. It shows that anxiety symptoms had a significant decrease in the estimate from pre-treatment to follow-up (Estimate=-5.78; SE = 0.57; df=53.44; *p*=<.001; 95% CI [-6.94, -4.62]). Regarding the secondary outcome, depressive symptoms, there was a significant decrease in estimate from pre-treatment to follow-up (Estimate=-4.02; SE = 0.64; df=59.51; *p*=<.001; 95% CI [-5.32, -2.72]). For the metacognition measurement, there was a significant negative estimate from pre-treatment to follow-up (Estimate=-19.43; SE = 2.37; df=73.94; *p*=<.001; 95% CI [-24.17, -14.69]).

### 3.5 Clinical significance

Reliable change is reported in **Table 5** for the primary outcome, GAD-7, and the secondary outcome, PHQ-9. Results indicated that 77.3% of the patients either recovered or improved using the GAD-7. Specifically, 54.7% of participants recovered at the end of treatment, and 22.6%. At booster, 55.3% qualified as recovered and 15.8% as improved.

**Table 5:** Clinical significance.

Clinical change	GAD-7		PHQ-9	
	Pre-Post	Pre-FollowUp	Pre-Post	Pre-FollowUp
	N=53	N=38	N=33	N=25
Recovered	N=29(54.7%)	N=21(55.3%)	N=5(15.2%)	N=7(28.0%)
Improved	N=12(22.6%)	N=6(15.8%)	N=6(18.2%)	N=3(12.0%)
Unchanged	N=12(22.6%)	N=11(28.9%)	N=19(57.6%)	N=14(56.0%)
Deteriorated	N=0(0%)	N=0(0%)	N=3(9.1%)	N=1(4.0%)

GAD-7, generalized anxiety disorder-7(primary outcome); PHQ-9, patient health questionnaire-9(secondary outcome); norm used for GAD-7 minimal detectable change ±4, range of normal functioning <8; PHQ-9 minimal detectable change ±6, range of normal functioning <7. For statistical estimation the participants below cut-off at pre-treatment for both GAD-7(<10) and PHQ-9(<10) were not included.

For the secondary outcome, which was not specifically targeted in treatment, 33.4% of those having a depression problem experienced a clinically significant change. Specifically, on the PHQ-9, 15.2% qualified as recovered at the end of treatment, and 18.2% as improved. At booster, 28.0% were recovered and 12.0% improved.

### 3.6 Sensitivity analysis

We conducted an additional paired sample t-test, excluding the six patients who received treatment despite having a GAD-7 score below cut-off. As presented in **Table 6**, these results show that findings are clinically significant and that effect sizes are large in GAD-7 measures from pre- to post-treatment(t=12.84, Sig.=<.001, d=1.76, 95% CI [1.32, 2.19]), and pre-treatment to follow-up(t=8.72, Sig.=<.001, d=1.41, 95% CI [0.95, 1.86]).

**Table 6:** Sensitivity analysis. Paired sample t-test results.

Measure and time point	t	df	Sig. (two-tailed)	Cohen’s d	95%	CI
					Lower	Upper
GAD-7						
Pre-Post	12.84	52	<.001	1.76	1.32	2.19
Pre-FollowUp	8.72	37	<.001	1.41	0.95	1.86

GAD-7, generalized anxiety disorder-7(primary outcome); t, t-value; df, degrees of freedom; Sig., significance value (p-value); 95% CI, 95% confidence interval.

## 4 Discussion

This study adds to the limited evidence on psychological interventions for university students by examining the feasibility and associated symptom changes of an ongoing group metacognitive therapy (gMCT) program delivered to students in Oslo. Overall, the findings suggest that gMCT is a feasible intervention in this outpatient student setting, in accordance with established standards for reporting feasibility (27, 35). Regarding feasibility metrics, uptake was satisfactory, treatment retention was high with low dropout, and data collection efficiency was acceptable. Compared with a similar gMCT study for GAD by Haseth et al. (8), the rates are comparable: recruitment feasibility was 72% in Haseth et al. and 69% in the present study. Haseth et al. reported 100% retention, while retention in the current study was 95.3%. In conclusion, the current study and others on gMCT (4, 8, 24, 25) portray a treatment and a format that is generally well tolerated, with low dropout compared with the 19.7% average dropout rate reported in a meta−analysis for psychotherapy of adults (36).

### 4.1 Changes in anxiety and depression symptoms:

Associated outcome findings indicate statistically significant reductions in anxiety, depressive symptoms, and maladaptive metacognitive beliefs, with effect sizes ranging from moderate to large. Overall, 77.3% of participants improved or recovered after post-assessment. The primary outcome (GAD-7) showed a substantial and statistically significant decrease from pre- to post-assessment that was maintained at follow-up, reflecting a clinically meaningful reduction in anxiety symptoms. Using the Jacobsen method, 54.7% of participants were classified as ‘recovered’ on GAD-7 at post-treatment and 55.3% at follow-up; an additional 22.6% (post) and 15.8% (follow-up) were classified as ‘improved’. These clinical-change rates are broadly consistent with previous gMCT studies (8, 25), although some between-study differences exist. For example, Haseth et al. (8) reported higher recovery (87.0%), but their sample was smaller (n=23) and restricted to GAD. Similarly, a recent trial of generic gMCT in a Norwegian specialized mental health setting reported an initial recovery rate of 80% for depression and with recovery largely maintained across follow-up (24). The secondary outcome (PHQ-9) also demonstrated significant reductions during assessment and continued improvement at follow-up, despite the sample being recruited primarily on the basis of anxiety (GAD-7). While 33.4% of participants with a pre-existing depression problem reported clinically significant improvements, the majority of the sample remained clinically unchanged on the secondary outcome.

### 4.2 Changes in metacognitions and worry

Metacognitive beliefs (MCQ-30) showed large and sustained decreases from pre- to post-assessment and follow-up (post d=1.34; follow-up d=1.04). These changes are consistent with the hypothesized mechanism of MCT-modifying maladaptive metacognitive processes associated with worry and rumination. CAS-1 scores recorded during assessment showed improvement across session measures, with a large effect size that persisted at follow-up measures. The present results are consistent with the theoretical model of MCT and that gMCT produces meaningful changes in the cognitive processes that maintain worry.

### 4.3 Theoretical and practical implications

These results can be interpreted as compatible with MCT’s transdiagnostic rationale: targeting metacognitive beliefs and processes can produce improvements across different anxiety disorders (3, 6, 37). Importantly, the outcomes are consistent with gMCT being a viable group format for delivering MCT principles - an implication of practical and theoretical interest given the currently limited research on group delivery. These findings also have important implications for university counselling services, given the substantial unmet need for mental health care among students, suggesting that metacognitive group therapy may represent a feasible and effective treatment option for students experiencing anxiety and depressive symptoms. Delivering MCT in a group format could increase service capacity while preserving treatment quality and producing clinically meaningful outcomes. Student welfare services might therefore consider implementing gMCT as part of a stepped-care approach, pending further confirmatory research.

### 4.4 Limitations

Several limitations warrant caution when interpreting the findings of this study, and the results should be considered preliminary and exploratory in nature. The study used an uncontrolled pre–post design without an experimental control group, which precludes causal inference; observed improvements may partly reflect natural recovery, placebo effects, natural symptom fluctuations, regression to the mean, or non−specific treatment effects. Furthermore, the sample size and the rate of attrition should also be noted. While the study began with N = 61 at baseline, the sample decreased to N = 59 at post-treatment and further to N = 43 at follow-up. This reduction at the follow-up stage raises concerns regarding attrition bias. When estimating clinical change for the GAD-7 and PHQ-9, participants who scored below the clinical cut-off at baseline were excluded. Using this subsample reduced the available data for analysis, particularly for the secondary outcome (PHQ-9), which may influence the robustness of those clinical estimates. Although sensitivity analyses indicated that including the six participants who scored below the GAD-7 cut-off at baseline did not materially change the primary results, the reduced subsample for clinical significance requires careful interpretation. The gender imbalance with a high female participation limits generalizability to the broader student population. This may be a reflection of help-seeking behavior differences between the genders. Even though the therapists in this study did receive supervision from an experienced MCT-therapist and followed a standardized treatment manual, methodological weaknesses include a lack of formal adherence checks to evaluate treatment fidelity. Finally, single−site delivery limits generalizability. Due to these limitations, caution is recommended when generalizing these findings to other settings or populations. To establish causality and clarify the added value of the group format, randomized controlled trials comparing gMCT to individual MCT, other active treatments, or waitlist controls are needed. Larger, multicenter trials with stratified sampling, gender balance, longer follow−up, as well as session-level process measures and fidelity monitoring would improve precision and external validity. Process studies examining session−by−session CAS trajectories and mediators of change would further clarify mechanisms and inform optimization of group protocols.

## 5 Conclusion

This study aimed primarily to investigate feasibility and associated preliminary symptom changes in routine care. Results showed that the intervention was feasible and that gMCT was associated with statistically and clinically meaningful reductions in worry, anxiety, depressive symptoms, and maladaptive metacognitive beliefs in this student sample. These findings add to the growing literature on gMCT and suggest that group delivery may represent a promising, scalable option for student mental health services. Controlled trials are recommended and needed to confirm these results and determine the comparative effectiveness and cost-efficiency of group versus individual MCT.

## Data Availability

The raw data supporting the conclusions of this article are not publicly available due to ethical and legal restrictions related to Norwegian data protection regulations. Data may be made available upon reasonable request to the corresponding author, subject to approval from the appropriate Regional Committee for Medical and Health Research Ethics in Norway.
